# Monoclonal antibodies for imaging and therapy.

**DOI:** 10.1038/bjc.1989.32

**Published:** 1989-02

**Authors:** A. A. Epenetos, C. Kosmas

**Affiliations:** Imperial Cancer Research Fund Oncology Group, Royal Postgraduate Medical School, Hammersmith Hospital, London, UK.


Br. J. Cancer (1989), 59, 152–155 © The Macmillan Press Ltd., 1989

### Monoclonal antibodies for imaging and therapy

A.A. Epenetos & C. Kosmas

*Imperial Cancer Research Fund Oncology Group, Royal Postgraduate Medical School, Hammersmith Hospital, Du Cane Road, London W12 0HS, UK.*

We are now in a scientific era where advances in basic understanding of cellular behaviour and technological breakthroughs are opening new and exciting avenues in cancer research and clinical management of patients with malignant disease. One such advancement is the development of monoclonal antibodies (Kohler & Milstein, 1975). Like many other 'breakthroughs' in biomedical research, monoclonal antibodies have been through the three phases of maturation. At the beginning, there was the excitement and hype that not only would monoclonal antibodies revolutionise the diagnosis, but they would also cure many, if not most, so far incurable cancers. In fact, early pilot studies had suggested that this could be the case in both diagnosis (Mach *et al.*, 1981; Epenetos *et al.*, 1982*a*; Farrands *et al.*, 1982) and therapy (Sears *et al.*, 1982; Miller *et al.*, 1982). Unfortunately this euphoria was short lived because subsequent and more comprehensive studies (Epenetos *et al.*, 1986) demonstrated a major fundamental problem with antibody targeting. That was, that after intravenous administration of monoclonal antibodies in patients, only a very small proportion of the injectate localised to tumour sites ($\sim$0.005% of the injected amount per gram of tumour) which is approximately 100–1,000 times less than when the same antibody is tested in animal models, e.g. nude mice bearing human cancer xenografts (Epenetos *et al.*, 1982*b*; Schlom, 1986). These small amounts of antibodies are hardly sufficient to allow successful imaging of small lesions undiagnosed by existing conventional radiological techniques and are certainly not sufficient to deliver tumoricidal amounts of cytotoxic agents to tumour cells without damaging other normal organs such as the bone marrow, liver and kidneys.

So what is the state of the art with monoclonal antibodies today? It is the author's opinion that we have now entered the third and, it is hoped, final phase of maturity, which is understanding the shortcomings of monoclonal antibodies and designing methods to overcome them. To understand the mechanism of antibody targeting to tumour tissues *in vivo*, at least two aspects need to be considered.

The first is the host handling of the antibody, which includes both normal and neoplastic tissues. After an antibody is injected into the blood stream the following events happen before it can reach the tumour cells (Jain, 1987): the antibody is diluted by the vascular volume; transported across microvessel endothelial cells; enters normal and neoplastic interstitium and binds on to specific antigens and possible internalisation.

Data available so far (Jain, 1987) indicate that the interstitial space of neoplastic tissues differs significantly from that of normal tissues both in structure and function. In general, tumours have high interstitial fluid pressure and fluid flow, probably due to the fact that their interstitial spaces are larger than normal and lack a functioning lymphatic system. This may be an advantage in using monoclonal antibodies for tumour targeting because a high intratumoral interstitial fluid pressure and flow may allow for more efficient interstitial diffusion of macromolecules such as antibodies. On the other hand, in large tumours high interstitial pressure and low microvessel pressure may slow down the extravasation of antibodies into tumour tissues. Of course, before an antibody reaches the interstitium of tumours it may be catabolised. Immunoglobulin catabolism is carried out by the reticuloendothelial system and the liver in particular, where both parenchymal and non-parenchymal cells are involved (Sands *et al.*, 1989).

The second aspect is the characteristics of individual monoclonal antibodies and their conjugates. Monoclonal antibodies, like all antibodies, consist of the minimum essential part required to bind antigen, i.e. the antigen binding loops of the V domains coded by the complementarity determining regions (CDRs) of the V genes and the rest of the V domains as well as constant regions of heavy and light immunoglobulin chains.

The exquisite specificity of monoclonal antibodies for their targets is determined by the 3-D structure of the V domain and CDR loops. The presence of tumour associated or tumour 'specific' antigens is an essential prerequisite for successful tumour targeting using antibodies. It is not the purpose of this article to review human tumour antigens but, rather, to focus on a few interesting examples which may prove useful in our selection of antigen–antibody systems.

### Human milk fat globule membrane

A highly immunogenic component of the human milk fat globule membrane is a large molecular weight mucin (>400 kD) which is also secreted into milk (Ceriani *et al.*, 1982). A large number of tumour associated antigens defined by existing monoclonal antibodies are carried out on molecules that are immunologically related or identical to the milk mucins (Taylor-Papadimitriou *et al.*, 1981; Arklie *et al.*, 1981). Some tumour mucins are aberrantly or incompletely glycosylated, allowing for their protein core to be exposed. This observation has been exploited and new monoclonal antibodies directed against protein core components of tumour mucins have been produced (Burchell *et al.*, 1987; Gendler *et al.*, 1987). Antibodies such as these, reacting with protein determinants which are only exposed in tumours, can be described as 'operationally tumour specific'.

In fact, from the vast pool of existing antitumour monoclonal antibodies, there exists a small group of antibodies directed against unique epitopes of antigens that allow for increased tumour specificity. These include the SM3 (Burchell *et al.*, 1987; Gendler *et al.*, 1987) against an epitope of stripped mucin and found widely on carcinomas, B72.3 (Hand *et al.*, 1985) against a glycoprotein on many tumours and OVTL3 (Poels *et al.*, 1986) and MOV 18 (Miotti *et al.*, 1987) against ovarian cancer.

### Multiple drug resistance proteins

It is possible that the ultimate place of monoclonal antibodies against cancer would be in conjunction with other anticancer agents such as chemotherapy. In that case it would be desirable and complementary if a monoclonal antibody would target against chemotherapy-resistant cancer

Correspondence: A.A. Epenetos.

cells. Studies of multiple drug resistant mutant cells have shed new light into drug transport in mammalian cells.

It would appear that simultaneous resistance to a wide range of unrelated chemotherapy and other drugs is mediated, at least in part, by an increase in a plasma membrane glycoprotein (p-glycoprotein, 170 kD) which acts like a molecular pump exporting a variety of compounds, including many chemotherapy drugs (Kartner *et al.*, 1985). Several monoclonal antibodies now exist that are directed against p-glycoprotein (Sugawara *et al.*, 1988). These may prove useful in *in vivo* therapeutic applications.

**Tumour microvasculature**

Another interesting antigenic target for tumour therapy may be the microvasculature of neoplastic tissues. If one could target selectively and destroy the cells forming tumour microvessels (without affecting, at the same time, normal tissue microvessels) then a significant antitumour effect could be achieved. In fact, recent reports (Watkins *et al.*, 1989) have indicated that some monoclonal antibodies originally made against malignant gliomas may target specifically to tumour microvessels.

Most clinical trials of *in vivo* diagnosis and therapy have involved either mouse or rat monoclonal antibodies. A crucial and yet not unexpected problem that has been identified when using rodent antibodies in humans is the sensitisation of the recipient to the administered xenogeneic protein (Schroff *et al.*, 1985; Courtenay-Luck *et al.*, 1986). Immunisation of patients would cause immune complex disease and also abrogate any therapeutic effect due to rapid clearance of administered antibody.

There are at least four ways that attempt to overcome this problem. The first is to use existing rodent antibodies but to delay and reduce the intensity of human antimouse antibody responses by the use of immunosuppressive agents such as cyclosporin A cyclophosphamide and steroids (Ortho Multicenter Transport Study Group, 1985; Begent, 1989). The second approach is to try and induce specific unresponsiveness to rodent immunoglobulins by the use of immunosuppressive antibodies (Benjamin & Waldmann, 1986) or by coupling substances such as polyethylene glycol, which apparently may convert xenogeneic monoclonal antibodies to specific tolerogens (Sehon, 1989). A third way is to use human monoclonal antibodies produced from Epstein-Barr virus transformed human b-lymphocytes or from human or human mouse hybridomas (Sikora *et al.*, 1982; Cote *et al.*, 1983). This approach has only had limited success so far but a new technique exploiting the immunological idiotypic network (Ritter *et al.*, 1987) may yield high affinity and high specificity human monoclonal antibodies. Finally, by using recombinant DNA technology it is now possible to engineer antibodies where only the antigen binding site is defined by mouse gene sequences and the rest of the molecule is 'human' (Riechmann *et al.*, 1988).

As stated earlier, the fundamental difficulty with antibody targeting to tumours is the small amount of immunoglobulin that reaches the tumour. Are there any ways to overcome this problem? One possibility is to use smaller fragments of the antibody molecule. It was thought that the large molecular weight of the intact IgG might be responsible for its poor accessibility to solid tumour tissue. It has been shown, however, that the use of either monomeric (Fab) or dimeric $(Fab')_2$ fragments does not achieve higher amounts of antibody in the tumour (Wahl *et al.*, 1983). Fragments produce faster and better tumour to non-tumour ratios because they are cleared more rapidly from the circulation and there is less non-specific uptake by Fc binding cells or carbohydrate catabolising cells (Sands *et al.*, 1989). A recent development has been the recombinant production of Fv fragments. These are the smallest possible intact fragments of the antibody molecule that can bind antigen in the appropriate configuration (Verhoeyen *et al.*, 1988). Fv fragments may prove superior in tumour targeting although their small size ($\sim$25 kD) may lead to even faster clearance.

A second possibility to increase tumour uptake of antibody is to give a much higher amount of antibody as long as this is relatively non-toxic to the host. It would be advantageous if the cytotoxic moeity or imaging isotope could then be delivered at a time when the tumour to non-tumour ratios are optimal and at a time when there was no significant circulating amount of antibody. Consideration of the difficulties of delivering adequate quantities of antibody has resulted in several current lines of research. Hybrid antibodies with dual specificities can be produced by the cell fusion of two Ig producing cell lines (Milstein & Guello, 1983; Clark & Waldmann, 1989) or by chemically conjugating two monomeric Fab fragments (Perez *et al.*, 1985). Hybrid antibody may be capable of targeting diagnostic or therapeutic radioisotopes such as indium-111 and yttrium-90 (Goodwin, 1987) or targeting drug (Corvalan *et al.*, 1988) or toxins (Webb *et al.*, 1985) to cells expressing appropriate cell surface antigens. Of greater interest, perhaps, may be the ability of these bispecific antibodies to induce potent tumour cell killing by activated T-cells if the bispecific antibody can cross-link T-cells and antigens on the surface of tumour cells (Clark & Waldman, 1989; Clark *et al.*, 1988; Canevari *et al.*, 1988).

Another novel approach is the *in vivo* use of streptavidin conjugated antibodies followed, after an appropriate period of time, by radioactive biotin either for imaging or therapy. Biotin has an extremely high affinity for streptavidin ($Dd = 15\,M^{-10}$) and at the same time is a small enough molecule that can diffuse rapidly through most tissues in the body. Using, initially, experimental models (Hnatowich *et al.*, 1987; Paganelli *et al.*, 1988) and more recently clinical studies (Hnatowich *et al.*, 1989) it has been shown that the tumour target to non-target ratios may be improved considerably.

A different and rather interesting strategy has recently been proposed by Professor K. Bagshawe and his colleagues, who used a two-step approach (Bagshawe *et al.*, 1989). In their system a prodrug is activated at the tumour site by a non-mammalian enzyme conjugated on to antibody. As the first step, the antibody–enzyme conjugate is injected and allowed to localise to tumours. After a certain period of time, it is followed by a prodrug which diffuses readily through tissues. This prodrug is relatively stable and only gets activated by the targeted enzyme on tumour sites. The prodrug becomes a highly toxic agent which can enter tumour cells and at the same time has a short half-life so that it is of low toxicity to normal tissues (Bagshawe *et al.*, 1989).

We have already acknowledged the difficulties resulting from the immunogenicity of rodent monoclonal antibodies when injected repeatedly into humans. This problem may be reduced but may not be totally eliminated even if one could use human monoclonal antibodies because of the possibility of developing anti-idiotypic and anti-allotypic antibodies (Ritter *et al.*, 1987). Can one, however, use this phenomenon to the patient's advantage? It has been shown by several workers (Schroff *et al.*, 1985; Courtenay-Luck *et al.*, 1986; Ritter *et al.*, 1987) that antigen antibody response developing in recipients is partly directed against the variable region (idiotype) of the administered antibody (anti-id[1] response). Some of these anti-idiotypic antibodies are directed against the combining site (paratope) of the administered monoclonal antibody and therefore represent an 'internal image' of the tumour antigen. In some cases, this can lead to a marked elevation of human serum antibodies that themselves bind to tumour antigen, with a specificity similar to that of the injected antibody. These are now human anti-tumour antibodies which may mediate a cytotoxic response. The discovery of anti-idiotypic antibodies has led to the proposal of making idiotypic vaccines in cases where purified tumour antigen is not available or is presented to the immune system in a non-immunogenic way. In fact animal studies using tumour specific idiotypic vaccines have produced encouraging results (Dunn *et al.*, 1987; Raychaudhari *et al.*, 1987). Therefore, the effects of generating anti-idiotypic responses may not necessarily be detrimental to the host. It may be possible to switch on such a response to the patient's advantage once we know how to manipulate the idiotypic network (Jerne, 1971) and provide the patient with auto-anti-tumour antibodies.

Some new concepts such as new tumour targets, recombinant antibodies and fragments, two-step strategies and exploitation of the idiotypic network have been briefly reviewed. In addition, there has been a great deal of progress in the basic chemistry of immunoconjugates providing increased stability and retention of immunoreactivity in antibody–drug conjugates (Kanellos *et al.*, 1985), immunotoxins (Thorpe *et al.*, 1987), antibody-isotopes (Moi *et al.*, 1985; Gansow *et al.*, 1989; Eaton *et al.*, 1989; Abrams *et al.*, 1989) and site specific radiolabelling (Rodwell *et al.*, 1986). We are only at the beginning of fully evaluating the potency of antibodies in cancer diagnosis and therapy. Some of the main issues involved in this approach are discussed in the proceedings of the Fifth International Meeting entitled 'Advances in the Application of Monoclonal Antibodies in Clinical Oncology' and published in this issue of the *British Journal of Cancer*.

### References

ABRAMS, P.G., SCHROFF, R.W., FER, M.F. & 5 others (1989). Successful imaging of metastatic tumours using labelled monoclonal antibodies: A prelude to therapy. *Br. J. Cancer*, **59** (in the press).

ARKLIE, J., TAYLOR-PAPADIMITRIOU, J., BODMER, W.F., EGAN M. & MILLIS, R. (1981). Differentiation antigens expressed by epithelial cells in the lactating breast are also detectable in breast cancers. *Int. J. Cancer*, **28**, 23.

BAGSHAWE, K.D., SEARLE, F., SPRINGER, C. & 4 others (1989). Tumour site activation of cytotoxic agent. *Br. J. Cancer*, **59** (in the press).

BEGENT, R.H.J. (1989). Dosimetry and suppression of the antimouse response in 131-iodine labelled antibody therapy of cancer. *Br. J. Cancer*, **59** (in the press).

BENJAMIN, R.J. & WALDMANN, H. (1986). Induction of tolerance by monoclonal antibody therapy. *Nature*, **320**, 449.

BURCHELL, J., GENDLER, S., TAYLOR-PAPADIMITRIOU, J. & 4 others (1987). Development and characterization of breast cancer reactive monoclonal antibodies directed to the core protein of the human milk fat globule. *Cancer Res.*, **47**, 5476.

CANERVARI, S., MENARD, S., MEZZANZANICA, D. & 4 others (1988). Anti-ovarian carcinoma anti-T3 heteroconjugates or hybrid antibodies induce tumour cell lysis by cytotoxic T-cells. *Int. J. Cancer*, Suppl. 2, 18.

CERIANI, R.L., SASAKI, M., SUSSMAN, H., WARA, W. & BLANK, E.W. (1982). Circulating human mammary epithelial antigens in breast cancer. *Proc. Natl Acad. Sci. USA*, **79**, 5420.

CLARK, M., GILLILAND, L. & WALDMANN, H. (1988). The potential of hybrid antibodies secreted by hybrid-hybridomas in tumour therapy. *Int. J. Cancer*, Suppl. 2, 15.

CLARK, M. & WALDMANN, H. (1989). Therapy with hybrid antibodies. *Br. J. Cancer*, **59** (in the press).

CORVALAN, J.R.F., SMITH, W. & GORE, V.A. (1988). Tumour therapy with vinca alkaloids targeted by a hybrid-hybrid monoclonal antibody recognising both CEA and vinca alkaloids *Int. J. Cancer*, Suppl. 2, 22.

COTE, R.J., MORRISSEY, D.M., HOUGHTON, A.N., BEATTIE, E.J., OETTGEN, H.F. & OLD, L.J. (1983). Generation of human monoclonal antibodies reactive with cellular antigens. *Proc. Natl Acad. Sci. USA*, **80**, 2026.

COURTENAY-LUCK, N.S., EPENETOS, A.A., MOORE, R. & 4 others (1986). Development of primary and secondary immune responses to mouse monoclonal antibodies used in the diagnosis and therapy of malignant neoplasms. *Cancer Res.*, **46**, 6489.

DUNN, P.L., JOHNSON, C.A., STYLES, J.M., PEASE, S.S. & DEAN, C.J. (1987). Vaccination with syngeneic monoclonal anti-idiotype protects against a tumour challenge. *Immunology*, **137**, 1743.

EATON, M.A.W., PARKER, D. & HARRISON, A. (1989). The use of macrocyclic ligands in radioimmunoconjugates. *Br. J. Cancer*, **59** (in the press).

EPENETOS, A.A., BRITTON, K.E., MATHER, S. & 8 others (1982*a*). Targetting of $^{123}$I-labelled tumour associated monoclonal antibodies to ovarian, breast and gastrointestinal tumours. *Lancet*, **ii**, 999.

EPENETOS, A.A., NIMON, C.C., ARKLIE, J. & 4 others (1982*b*). Radioimmunodiagnosis of human cancer in an animal model using labelled tumour associated monoclonal antibodies. *Br. J. Cancer*, **46**, 1.

EPENETOS, A.A., SNOOK, D., DURBIN, H., JOHNSON, P.M. & TAYLOR-PAPADIMITRIOU, J. (1986). Limitation of radiolabelled monoclonal antibodies for localisation of human neoplasms. *Cancer Res.*, **46**, 3183.

FARRANDS, P.A., PERKINS, A.C., PIMM, M.V., BALDWIN, R.W. & HARDCASTLE, J.D. (1982). Radio-immunodetection of human colorectal cancers using an anti-tumour monoclonal antibody. *Lancet*, **i**, 397.

GANSOW, O.A., BRECHBIEL, M.W., KUMAR, K., MIRZADEHS, S., McMURRAY, T. & MAGERSTADT, M. (1989). Polyazamacrocyclic chelating agents for radioimmunoimaging and radioimmunotherapy. *Br. J. Cancer*, **59** (in the press).

GENDLER, S.J., BURCHELL, J.M., DUHIG, T., WHITE, R., PARKER, M. & TAYLOR-PAPADIMITRIOU, J. (1987). Cloning the cDNA coding for the differentiation and tumour associated mucin glycoproteins expressed by human mammary epithelium. *Proc. Natl Acad. Sci. USA*, **84**, 6060.

GOODWIN, D.A. (1987). Pharmacokinetics and antibodies. *J. Nucl. Med.*, **28**, 1358.

HAND, P.H., COLCHER, D., SALOMON, D., RIDGE, J., NOGUCHI, P. & SCHLOM, J. (1985). Influence of spatial configuration of carcinoma cell populations on the expression of a tumour-associated glycoprotein. *Cancer Res.*, **45**, 833.

HNATOWICH, D.J., ROWLINSON, G., RUSKOWSKI, M., SNOOK, D. & EPENETOS, A.A. (1989). Tumour localisation studies with streptavidin and biotin. *Br. J. Cancer*, **59** (in the press).

HNATOWICH, D.J., VIRZI, F. & RUSCKOWSKI, M. (1987). Investigations of avidin and biotin for imaging applications. *J. Nucl. Med.*, **28**, 1294.

JAIN, K.R. (1987). Transport of molecules in the tumour interstitium: A review. *Cancer Res.*, **47**, 3039.

JERNE, N.K. (1971). Towards a network theory of the immune system. *Ann. Immunol.*, **125C**, 373.

KANELLOS, J., PIETERSZ, G.A. & McKENZIE, I. (1985). Studies of methotrexate monoclonal antibody conjugates for immunotherapy. *J. Natl Cancer Inst.*, **75**, 319.

KARTNER, N. EVENDEN-PORELLE, D., BRADLEY, G. & LING, V. (1985). Detection of P-glycoprotein in multi-drug resistant cell lines by monoclonal antibodies. *Nature*, **316**, 820.

KOHLER, G. & MILSTEIN, C. (1975). Continuous culture of fused cells secreting antibody of predefined specificity. *Nature*, **248**, 465.

MACH, J.P., BUCHEGGER, F., FORNI, M. & 7 others (1981). Use of radiolabelled monoclonal anti-CEA antibodies for the detection of human carcinomas by external photoscanning and tomoscintigraphy. *Immunol. Today*, **2**, 239.

MILLER, R.A., OSEROFF, A.R., STRATTE, P.T. & LEVY, R. (1982). Treatment of B-cell lymphoma with monoclonal anti-idiotype antibody. *N. Engl. J. Med.*, **306**, 517.

MILSTEIN, C. & GUELLO, C. (1983). Hybrid-hybrid myelomas and their use in immunohistochemistry. *Nature*, **305**, 537.

MIOTTI, S., CANEVARI, S., MENARD, S. & 6 others (1987). Characterization of human ovarian carcinoma-associated antigens defined by novel monoclonal antibodies with tumour restricted specificity. *Int. J. Cancer*, **39**, 297.

MOI, M.K., MEARES, C.G., McCALL, M.J., COLE, W.C. & DeNARDO, S.J. (1985). Copper chelates as probes of biological systems: Stable copper complexes with a macrocyclic bifunctional chelating agent. *Analyt. Biochem.*, **148**, 249.

ORTHO MULTICENTER TRANSPORT STUDY GROUP (1985). A randomised clinical trial of OKT3 monoclonal antibody for acute rejection of cadaveric renal transplants. *N. Engl. J. Med.*, **313**, 337.

PAGANELLI, G., RIVA, P., DELEIDE, G. & 5 others (1988). *In vivo* labelling of biotinylated monoclonal antibodies by radioactive avidin: A strategy to increase tumour radiolocalisation. *Int. J. Cancer*, Suppl. 2, 121.

PEREZ, P. HOFFMANN, R.W., SHAW, S., TITUS, J.A. & SEGAL, D.M. (1985). Specific targeting of cytotoxic T-cells by anti-T3 pinned to anti-target cell antibody. *Nature*, **316**, 354.

POELS, L.G., PETERS, D., VAN MEGEN, Y. & 7 others (1986). Monoclonal antibody against human ovarian tumour-associated antigens. *J. Natl Cancer Inst.*, **76**, 781.

RIECHMANN, L., CLARK, M., WALDMANN, H. & WINTER, G. (1988). Reshaping human antibodies for therapy. *Nature*, **332**, 323.

RITTER, M.A., COURTENAY-LUCK, N.S., SIVOLAPENKO, G.B. & EPENETOS, A.A. (1987). Human immune responses: The full network. *Br. J. Cancer*, **56**, 509.

SANDS, H., JONES, P.L., BROWN, B.A. & NASON, T. (1989). Uptake of radiolabel in rat liver cells after administration of radiolabelled B72.3 and its $F(ab)_2$ fragments. *Br. J. Cancer*, **59** (in the press).

SCHLOM, J. (1986). Basic principles and applications of monoclonal antibodies in the management of carcinomas. The Richard and Hinda Rosenthal Foundation Award Lecture. *Cancer Res.*, **46**, 3225.

SCHROFF, R.W., FOON, K.A., BEATTY, S.M., OLDHAM, R.K. & MORGAN, A.C. (1985). Human anti-murine immunoglobulin responses in patients receiving monoclonal antibody therapy. *Cancer Res.*, **45**, 879.

SEARS, H.F., ATKINSON, B., MATTIS, J., ERNST, C., HERLYN, D. & KOPROWSKI, H. (1982). Phase I clinical trial of monoclonal antibody in the treatment of gastrointestinal tumours. *Lancet*, **i**, 762.

SEHON, A.H. (1989). Conversion of xenogeneic monoclonal antibodies to specific tolerogens. *Br. J. Cancer*, **59** (in the press).

SIKORA, K., ALDERSON, T., PHILLIPS, J. & WATSON, J. (1982). Human hybridomas from malignant gliomas. *Lancet*, **ii**, 11.

SUGAWARA, I., KATOOKA, I., MORISHITA, Y., HAMOUDA, H. & TSUMO, T. (1988). Tissue distribution of P-glycoprotein encoded by a multi-drug-resistant gene as revealed by a monoclonal antibody MRK-16. *Cancer Res.*, **48**, 1926.

TAYLOR-PAPADIMITRIOU, J., PETERSON, J.A., ARLIE, J., BURCHELL, J., CERIANI, R.L. & BODMER, W.F. (1981). Monoclonal antibodies to epithelium specific components of the human milk fat globule membrane: Production and reaction with cells in culture. *Int. J. Cancer*, **28**, 17.

THORPE, P.E., WALLACE, P.M., KNOWLES, P.P. & 6 others (1987). New coupling agents for the synthesis of immunotoxins containing a hindered disulfide bond with improved stability *in vivo*. *Cancer Res.*, **47**, 5924.

VERHOEYEN, M., MILSTEIN, C. & WINTER, G. (1988). Reshaping human antibodies: Grafting an antilysozyme activity. *Science*, **239**, 1534.

WATKINS, B.A., CARTER, P., SCARAVILLI, F., DUCHEN, L.W. & THOMAS, D.G.T. (1989). Comparison of the specificity of monoclonal antibodies to malignant gliomas. *Br. J. Cancer*, **59** (in the press).

WEBB, K.S., WARE, J.L., PARKS, S.F., WALTHER, P.J. & PAULSON, D.F. (1985). Evidence for a novel hybrid immunotoxin recognising ricin-A-chain by one antigen-combining site and a prostate-restricted antigen by the remaining antigen combining site: Potential for immunotherapy. *Cancer Treat. Rep.*, **69**, 663.


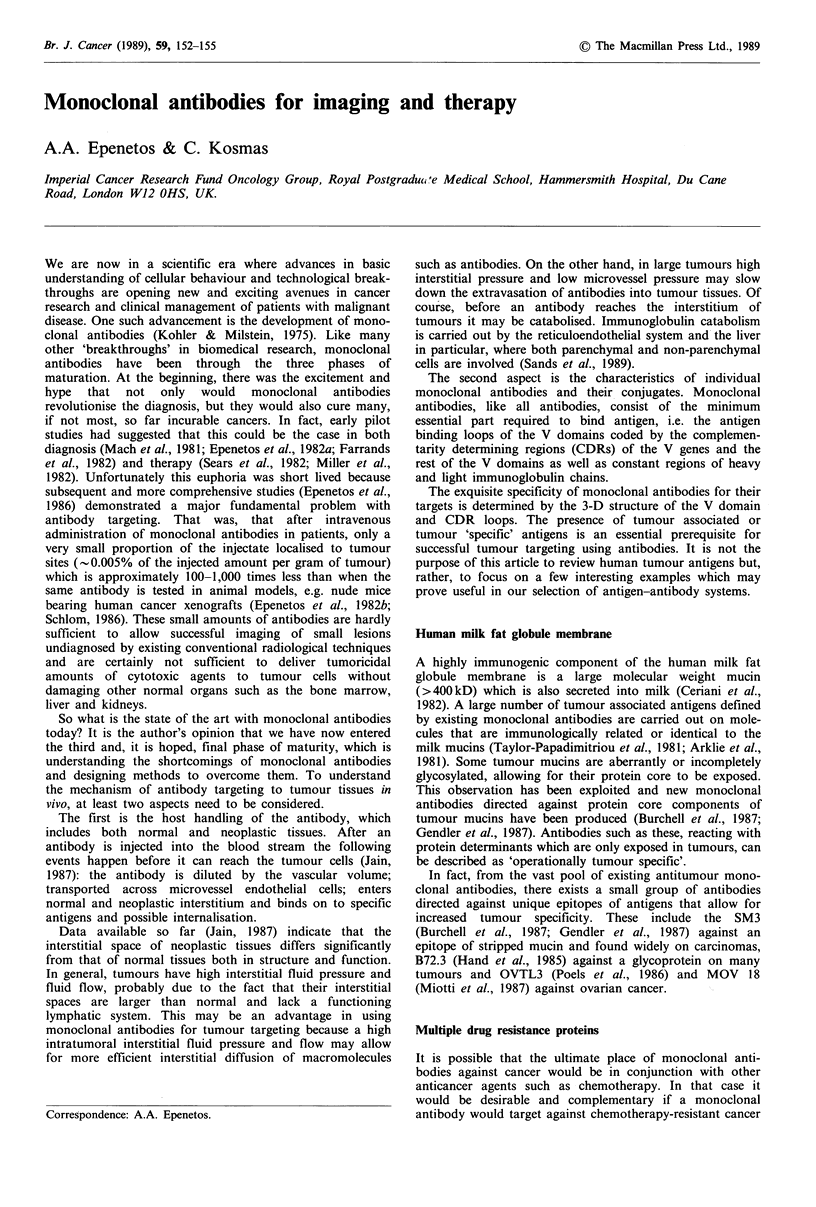

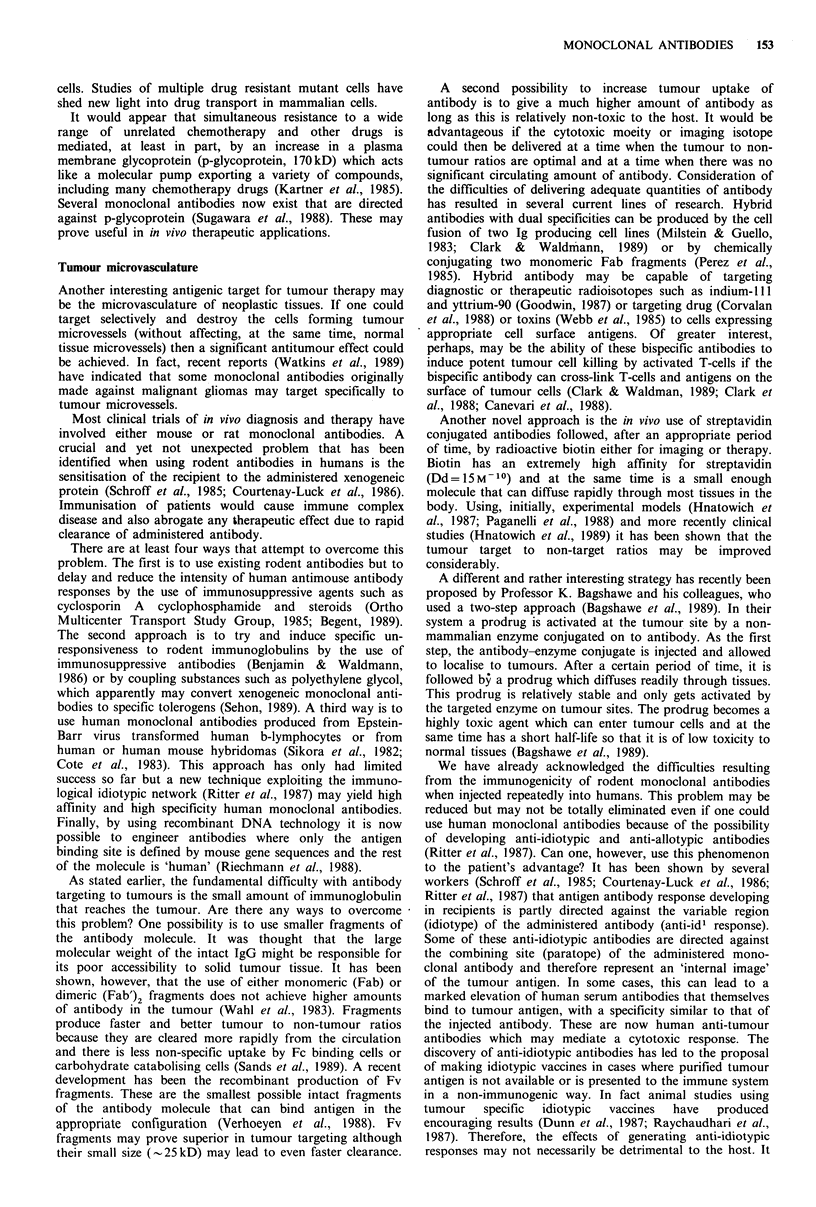

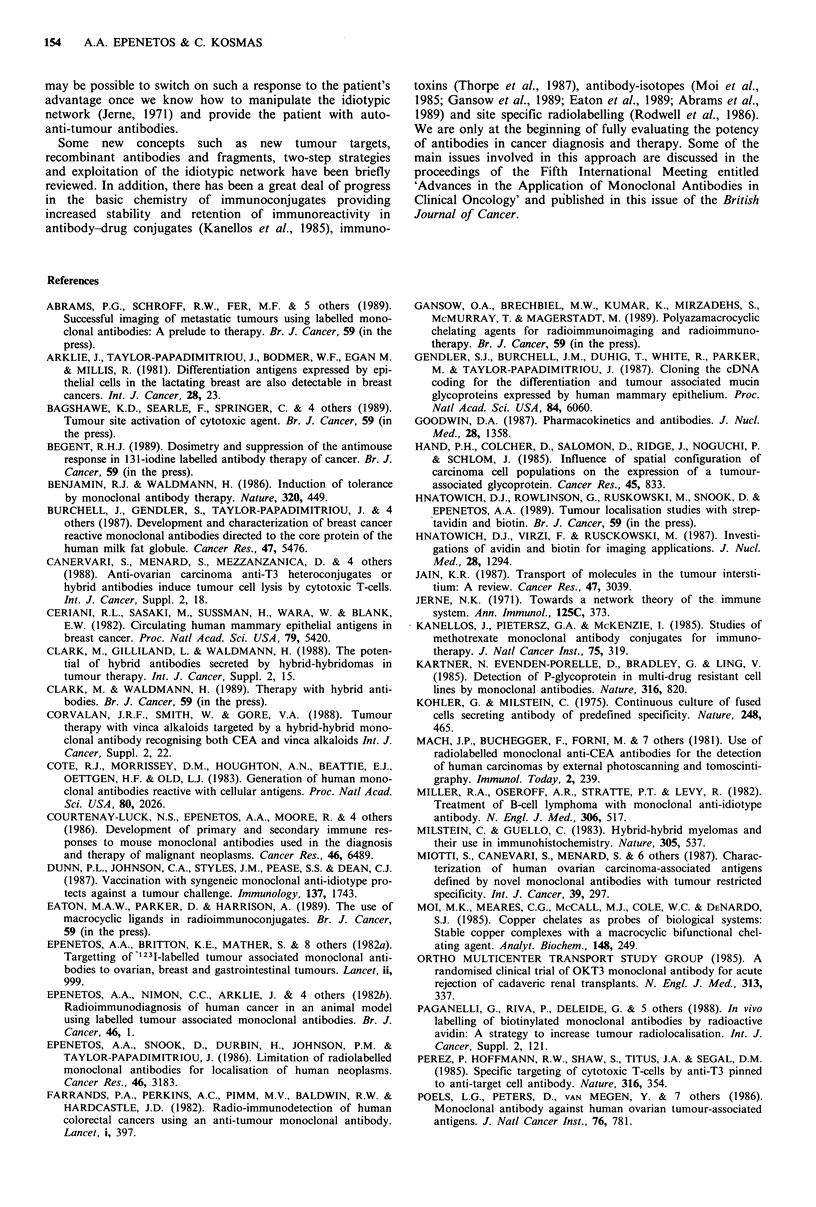

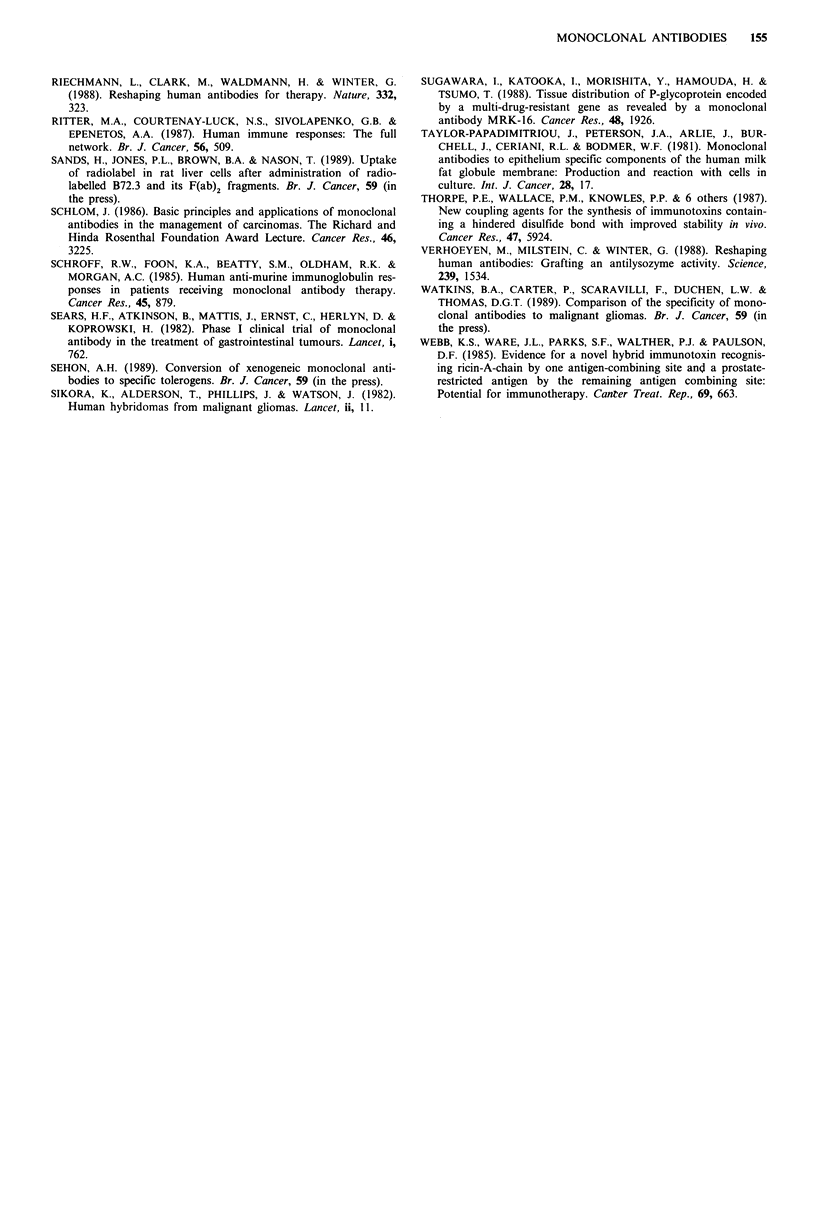


## References

[OCR_00548] (1987). Advances in the applications of monoclonal antibodies in clinical oncology. London, 6-8 May 1987. Abstracts.. Br J Cancer.

[OCR_00354] Benjamin R. J., Waldmann H. (1986). Induction of tolerance by monoclonal antibody therapy.. Nature.

[OCR_00358] Burchell J., Gendler S., Taylor-Papadimitriou J., Girling A., Lewis A., Millis R., Lamport D. (1987). Development and characterization of breast cancer reactive monoclonal antibodies directed to the core protein of the human milk mucin.. Cancer Res.

[OCR_00370] Ceriani R. L., Sasaki M., Sussman H., Wara W. M., Blank E. W. (1982). Circulating human mammary epithelial antigens in breast cancer.. Proc Natl Acad Sci U S A.

[OCR_00390] Cote R. J., Morrissey D. M., Houghton A. N., Beattie E. J., Oettgen H. F., Old L. J. (1983). Generation of human monoclonal antibodies reactive with cellular antigens.. Proc Natl Acad Sci U S A.

[OCR_00396] Courtenay-Luck N. S., Epenetos A. A., Moore R., Larche M., Pectasides D., Dhokia B., Ritter M. A. (1986). Development of primary and secondary immune responses to mouse monoclonal antibodies used in the diagnosis and therapy of malignant neoplasms.. Cancer Res.

[OCR_00424] Epenetos A. A., Snook D., Durbin H., Johnson P. M., Taylor-Papadimitriou J. (1986). Limitations of radiolabeled monoclonal antibodies for localization of human neoplasms.. Cancer Res.

[OCR_00430] Farrands P. A., Perkins A. C., Pimm M. V., Embleton M. J., Hardy J. D., Baldwin R. W., Hardcastle J. D. (1982). Radioimmunodetection of human colorectal cancers by an anti-tumour monoclonal antibody.. Lancet.

[OCR_00442] Gendler S. J., Burchell J. M., Duhig T., Lamport D., White R., Parker M., Taylor-Papadimitriou J. (1987). Cloning of partial cDNA encoding differentiation and tumor-associated mucin glycoproteins expressed by human mammary epithelium.. Proc Natl Acad Sci U S A.

[OCR_00449] Goodwin D. A. (1987). Pharmacokinetics and antibodies.. J Nucl Med.

[OCR_00464] Hnatowich D. J., Virzi F., Rusckowski M. (1987). Investigations of avidin and biotin for imaging applications.. J Nucl Med.

[OCR_00453] Horan Hand P., Colcher D., Salomon D., Ridge J., Noguchi P., Schlom J. (1985). Influence of spatial configuration of carcinoma cell populations on the expression of a tumor-associated glycoprotein.. Cancer Res.

[OCR_00469] Jain R. K. (1987). Transport of molecules in the tumor interstitium: a review.. Cancer Res.

[OCR_00473] Jerne N. K. (1974). Towards a network theory of the immune system.. Ann Immunol (Paris).

[OCR_00477] Kanellos J., Pietersz G. A., McKenzie I. F. (1985). Studies of methotrexate-monoclonal antibody conjugates for immunotherapy.. J Natl Cancer Inst.

[OCR_00482] Kartner N., Evernden-Porelle D., Bradley G., Ling V. Detection of P-glycoprotein in multidrug-resistant cell lines by monoclonal antibodies.. Nature.

[OCR_00498] Miller R. A., Maloney D. G., Warnke R., Levy R. (1982). Treatment of B-cell lymphoma with monoclonal anti-idiotype antibody.. N Engl J Med.

[OCR_00503] Milstein C., Cuello A. C. (1983). Hybrid hybridomas and their use in immunohistochemistry.. Nature.

[OCR_00507] Miotti S., Canevari S., Ménard S., Mezzanzanica D., Porro G., Pupa S. M., Regazzoni M., Tagliabue E., Colnaghi M. I. (1987). Characterization of human ovarian carcinoma-associated antigens defined by novel monoclonal antibodies with tumor-restricted specificity.. Int J Cancer.

[OCR_00513] Moi M. K., Meares C. F., McCall M. J., Cole W. C., DeNardo S. J. (1985). Copper chelates as probes of biological systems: stable copper complexes with a macrocyclic bifunctional chelating agent.. Anal Biochem.

[OCR_00525] Paganelli G., Riva P., Deleide G., Clivio A., Chiolerio F., Scassellati G. A., Malcovati M., Siccardi A. G. (1988). In vivo labelling of biotinylated monoclonal antibodies by radioactive avidin: a strategy to increase tumor radiolocalization.. Int J Cancer Suppl.

[OCR_00531] Perez P., Hoffman R. W., Shaw S., Bluestone J. A., Segal D. M. (1985). Specific targeting of cytotoxic T cells by anti-T3 linked to anti-target cell antibody.. Nature.

[OCR_00536] Poels L. G., Peters D., van Megen Y., Vooijs G. P., Verheyen R. N., Willemen A., van Niekerk C. C., Jap P. H., Mungyer G., Kenemans P. (1986). Monoclonal antibody against human ovarian tumor-associated antigens.. J Natl Cancer Inst.

[OCR_00543] Riechmann L., Clark M., Waldmann H., Winter G. (1988). Reshaping human antibodies for therapy.. Nature.

[OCR_00559] Schlom J. (1986). Basic principles and applications of monoclonal antibodies in the management of carcinomas: the Richard and Hinda Rosenthal Foundation award lecture.. Cancer Res.

[OCR_00565] Schroff R. W., Foon K. A., Beatty S. M., Oldham R. K., Morgan A. C. (1985). Human anti-murine immunoglobulin responses in patients receiving monoclonal antibody therapy.. Cancer Res.

[OCR_00571] Sears H. F., Atkinson B., Mattis J., Ernst C., Herlyn D., Steplewski Z., Häyry P., Koprowski H. (1982). Phase-I clinical trial of monoclonal antibody in treatment of gastrointestinal tumours.. Lancet.

[OCR_00581] Sikora K., Alderson T., Phillips J., Watson J. V. (1982). Human hybridomas from malignant gliomas.. Lancet.

[OCR_00585] Sugawara I., Kataoka I., Morishita Y., Hamada H., Tsuruo T., Itoyama S., Mori S. (1988). Tissue distribution of P-glycoprotein encoded by a multidrug-resistant gene as revealed by a monoclonal antibody, MRK 16.. Cancer Res.

[OCR_00593] Taylor-Papadimitriou J., Peterson J. A., Arklie J., Burchell J., Ceriani R. L., Bodmer W. F. (1981). Monoclonal antibodies to epithelium-specific components of the human milk fat globule membrane: production and reaction with cells in culture.. Int J Cancer.

[OCR_00598] Thorpe P. E., Wallace P. M., Knowles P. P., Relf M. G., Brown A. N., Watson G. J., Knyba R. E., Wawrzynczak E. J., Blakey D. C. (1987). New coupling agents for the synthesis of immunotoxins containing a hindered disulfide bond with improved stability in vivo.. Cancer Res.

[OCR_00604] Verhoeyen M., Milstein C., Winter G. (1988). Reshaping human antibodies: grafting an antilysozyme activity.. Science.

[OCR_00615] Webb K. S., Ware J. L., Parks S. F., Walther P. J., Paulson D. F. (1985). Evidence for a novel hybrid immunotoxin recognizing ricin A-chain by one antigen-combining site and a prostate-restricted antigen by the remaining antigen-combining site: potential for immunotherapy.. Cancer Treat Rep.

